# Ethik First – extracurricular support for medical students and young physicians facing moral dilemmas in hospital routine

**DOI:** 10.3205/zma001470

**Published:** 2021-04-15

**Authors:** Eva Kuhn, Laura Lunden, Penelope Moysich, Kai Rogge, Marijke Roscher, Lotta Caning, Annette Rogge

**Affiliations:** 1 University Hospital Bonn, Institute of Hygiene and Public Health, Section Global Health, Bonn, Germany; 2 UKSH Kiel, Clinic for Anesthesia and Operative Intensive Care Medicine, Kiel, Germany; 3 Christian Albrechts Universität zu Kiel, Kiel, Germany; 4 Fernuniversität Hagen, Hagen, Germany; 5 Helios Clinic Hildesheim, Pediatrics, Hildesheim, Germany; 6 Christian Albrechts Universität zu Kiel, Institute for Experimental Medicine, Medical Ethics, Kiel, Germany

**Keywords:** moral dilemma, moral distress, clinical ethics, problem-based learning

## Abstract

**Introduction:** Moral value conflicts play an increasingly central role in everyday hospital life. Clinical ethics, however, is only marginally represented in the compulsory curriculum for human medicine and the additional education regulations. The aim of the Ethik First project at the University Medical Center Schleswig-Holstein, Campus Kiel is to close this gap with an extracurricular offer and to support medical students from the fifth clinical semester onward and during their practical year as well as assistant doctors in dealing with moral dilemmas in everyday hospital life. The project has taken the concomitant learning objectives from the national competency-based learning objective catalog for medicine. According to the target group, the address in particular, showed higher taxonomy levels.

**Project description: **The multimodal concept is based on three pillars: In monthly principle-based case conferences, participants practice ethical reflection and moral judgment primarily on the basis of concrete cases introduced by them using the methods of problem-based learning and consideration-based deliberation. If participants do not bring forth a case, they discuss ethical aspects of current political relevance. Moreover, there is an annual public speaker event.

**Results: **Since the project began in 2017, ~20 students and interns have taken part in Ethik First one or more times. In a web-based interim evaluation (N=13), all respondents fully agreed that they considered the format helpful for dealing with ethical questions at the clinic. They rated the relevance for their later profession as high. There is evidence for support in moral dilemma situations.

**Discussion: **The first evaluation results of the voluntary extracurricular offer show the acceptance of the selected format, which goes beyond pure teaching in its conception in that it addresses moral stress as well and strengthens the participants’ individual resilience.

**Conclusion: **Ethik First reinforces the role of ethical aspects in the training of (prospective) doctors and focuses on reflecting on cases they have experienced firsthand.

We formulate a desideratum for appropriate advanced training concepts both in medical studies and in advanced medical training such that the training and development of comparable projects at medical faculties and at medical associations with student participation can be discussed.

## Zusammenfassung

**Einleitung: **Moralische Wertekonflikte spielen im Klinikalltag immer wieder und zunehmend eine zentrale Rolle. Klinische Ethik ist jedoch nur marginal im Pflichtcurriculum für Humanmedizin und den Weiterbildungsordnungen vertreten. Ziel des Projektes Ethik First am Universitätsklinikum Schleswig-Holstein, Campus Kiel ist es daher, diese Lücke mit einem extracurricularen Angebot zu schließen und Medizinstudierende ab dem 5. klinischen Semester sowie im Praktischen Jahr und Assistenzärzt*innen im Umgang mit moralischen Dilemmata im Klinikalltag zu unterstützen. Die damit einhergehenden Lernziele sind dem Nationalen Kompetenzbasierten Lernzielkatalog Medizin entnommen und adressieren der Zielgruppe entsprechend insbesondere höhere Taxonomiestufen.

**Projektbeschreibung: **Das multimodale Konzept beruht auf drei Säulen: Vorrangig werden in monatlich stattfindenden prinzipienorientierten Fallkonferenzen ethische Reflexion und moralisches Urteilen anhand konkreter von den Teilnehmer*innen eingebrachter Fälle nach den Methoden des problembasierten Lernens und der erwägungsorientierten Deliberation eingeübt. Wird durch die Teilnehmer*innen kein Fall eingebracht, werden alternativ ethische Aspekte von aktueller tagespolitischer Relevanz gemeinsam diskutiert. Ergänzend findet jährlich eine öffentliche Veranstaltung eines*einer Referenten*in statt.

**Ergebnisse: **Seit Beginn des Angebots im Jahr 2017 nahmen circa 20 Studierende und Assistenzärzt*innen ein- oder mehrmals an Ethik First teil. In einer webbasierten Zwischenevaluation (N=13) stimmten alle Befragten voll zu, dass sie das Format für hilfreich halten, um ethische Fragestellungen aus der Klinik zu bearbeiten. Die Relevanz für den späteren Beruf wurde hoch eingeschätzt. Es ergeben sich Anhalte für eine Unterstützung bei moralischen Dilemmasituationen.

**Diskussion: **Erste Evaluationsergebnisse des freiwilligen extracurricularen Angebotes zeigen die Akzeptanz des gewählten Formates, das in seiner Konzeption über reine Lehre hinaus geht, indem auch moralischer Stress adressiert wird und die individuelle Resilienz der Teilnehmer*innen gestärkt werden soll.

**Schlussfolgerung: **Ethik First stärkt die Rolle ethischer Aspekte in der Ausbildung von (angehenden) Ärzt*innen und stellt die Reflexion selbst erlebter Fälle in den Mittelpunkt.

Wir formulieren ein Desiderat an entsprechenden Fort- und Weiterbildungskonzepten sowohl im Medizinstudium als auch in der ärztlichen Weiterbildung, sodass Aus- und Aufbau vergleichbarer Angebote an medizinischen Fakultäten und auch an den Ärztekammern mit studentischer Beteiligung diskutiert werden sollten.

## 1. Introduction

### 1.1. Problem

Interns, students in their practical year (PY) and newcomers to the medical profession usually come with their own high standards of morally correct actions and behavior. Medical care, however, repeatedly confronts all actors in the health sector with situations that are characterized by moral uncertainty or that lead those involved into moral dilemmas. Strict hierarchies, institutional hurdles and economic constraints of the health system can make it difficult or impossible to follow one’s own values in patient care or to verbalize moral conflicts [1]. Examples are situations in which economic interests determine the therapy because of false incentives in the DRG system. A report on “Patient welfare as an ethical standard in hospitals” shows the fact that the German Ethics Council saw the requirement to emphasize the relevance and potential for conflict of economic aspects in German hospitals. It unanimously states that by “primarily focusing on reducing expenditure for health insurance companies and increased earnings on the part of the providers, effects emerged that give cause for concern with regard to patient well-being as a decisive normative benchmark” [2].

Such value conflicts cause moral stress, a term that was first introduced into the nursing sciences by the philosopher Andrew Jameton [3]; however, it is now intensively being researched for various health professions. In a broad definition, it describes psychological reactions to moral challenges [4]. In addition to a high workload and/or a lack of appreciation, moral stress is a factor that can lead to psychological overload and even stress-induced depression in young doctors [5], [6]. The experience of moral stress can lead to the intention to give up the medical profession [7].

With an average of 2.18 semester hours per week in 2014 [8], however, the curricular cross-sectional area “history, theory, ethics of medicine” (section 27, paragraph 1, sentence 5, no. 2 ÄApprO) can usually not address situations that may lead to moral stress within a reasonable time frame. The curriculum should address structural conditions that can lead to value conflicts and analyze them within its framework. Upon the entry into everyday clinical practice, however, a personal and immediate confrontation with value conflicts takes place, which necessitate additional retrospective reflection on one’s own experience [9]. The strong emotions that can accompany moral stress, especially feelings of guilt and shame, necessitate a safe setting to be able to reflect on what has been experienced [10]. Creating an intimate atmosphere is only possible to a limited extent in mandatory classes in the cross-sectional area because of the short time and the focus on imparting knowledge required for the examination.

Beyond the courses in the cross-sectional area of “history, theory, ethics of medicine”, medical professionals learn the so-called “hidden” and “informal curriculum” values and moral points of view [11]. "Hidden curriculum" indicates implicitly passed on social rules of a team or clinic, values inherent in the organizational and study structure and subconscious influences of the clinic culture [12]. The “informal curriculum” is closely interwoven and partly overlapping with this. To a large extent, this includes interpersonal communication in the team with patients and relatives as well as copying behavior patterns or the medical attitude of superiors. This type of learning and knowledge acquisition goes far beyond formalized bedside classes and explicit discussions [13]. Ethik First serves as a platform to point out these elements, which have not been considered to date in the formal curriculum, to recognize and name them as problematic, and to introduce them into the case discussion. 

In everyday clinical practice, moral dilemmas have to date been addressed in particular through projects from the clinical ethics committee (CEC) in the form of ethical case consultations, ethics consultations, and ethics ward visits [14]. They focus primarily on treatment decisions and patient care and not on doctors’ moral stress or only implicitly. In particular, ethical case consultations are often requested by or on the instruction of senior physicians, although they are also formally available to all employees. It is safe to assume that in these interdisciplinary meetings within the ward, interns, trainees and young professionals will find it difficult to verbalize their own values, particularly if these may not be congruent with those of their superiors. 

#### 1.2. Project aim 

The project aims to improve moral judgment, ethical reflection and strengthen individual resilience in value conflicts.

The central element is to give medical students in higher clinical semesters, trainees and junior doctors the opportunity to discuss and reflect on a moral dilemma they experience in everyday clinical life in a safe setting and under supervision. Such an exchange serves the (self-) reflection on one's own values and moral duties as (prospective) doctors, as well as the discussion of moral stress and aspects of the hidden and informal curriculum. 

Furthermore, networking of the participants of different training stages should be made possible and promoted. An overarching and long-term goal of the project is that (prospective) doctors get to know about the relevance of ethical topics in medicine and the work of the CEC from the start.

## 2. Project description

### 2.1. Concept

Ethik First was established in 2017 as an initiative of the managing director of the CEC of the University Medical Center Schleswig-Holstein, Campus Kiel and set up together in an interdisciplinary working group of four medical students and one theologian. It is anchored as an extracurricular support project and advanced training format in the clinical context in the field of ethics. Because of the dual target group, medical students in higher clinical semesters and assistant doctors, the project is embedded in the course and in the clinic. The dates will be announced on a homepage [http://www.ethik-first.de] and in an e-mail list of previous participants.

Since the beginning, the “UKSH Gutes tun!” Foundation of the Schleswig-Holstein University Medical Center has financially supported the project (e.g. for advertising) and remunerated external speakers.

#### 2.2. Organization and implementation

Ethik First comprises three pillars:

1. The focus is on monthly case conferences of 1 h duration each. Participants discuss cases that have been experienced by them and previously submitted for discussion. However, there is the possibility of expressing topic requests, which participants can then discuss using current patient cases or those described in the literature. They should take care, however, to discuss extreme/rare case constellations and regular ethical decisions and moral dilemmas that arise in everyday clinical practice [15]. This includes, for example, changes in therapy goals, the justification of mandatory measures, conflicts because of DRG requirements or difficult communication in the team and/or with relatives.
2. The second pillar relates to current ethical issues that affect society as a whole. These are discussed in the context of the monthly meetings and related in previous meetings, e.g., to non-invasive prenatal diagnostics (NIPD), triage in the pandemic or the resolution of contradictions in the case of post-mortem organ donation. This pillar is secondary to the first pillar, i.e., within the framework of the monthly Ethik First meeting, an issue that affects society as a whole is only discussed if no case has been submitted.
3. The third pillar comprises lectures by external speakers tailored to the focus group (e.g. on moral stress, ethics through the use of serious games). These take place once a year as planned and are open to all clinic employees and students.

#### 2.3. Didactic concept

Pillar 1 (case conferences): The learning objectives of Ethik First are the knowledge and application of technical and methodological skills and, based on this, the enhancement of the participants’ moral judgment. Table 1 (Tab. 1) shows the learning objectives for Ethik First corresponding to the National Competence-based Catalog of Learning Objectives for Medicine (NKLM) and their correlation to the learning objective taxonomy according to Anderson and Krathwohl [http://www.nklm.de] [16]. In line with the advanced training stage of the participants, Ethik First primarily addresses higher taxonomy levels.

Participants determine the professional skills to be separately imparted for each case discussion to align them as closely as possible with the patient case submitted. In terms of content, they use guidelines and position papers published by the professional associations as a basis, e.g., the position paper “Change of therapy goals and therapy limitation in intensive care medicine” of the Ethics section of DIVI [17], as well as the “decision-making aid for extended intensive care treatment needs on the way to organ donation” by DIVI [18].

 Through the two methods of problem-based learning [19], [20] and experience-based learning [21] in particular, Pillar 1 promotes the competencies of the participants. In the specific case discussion, the didactic method of conside ration-oriented deliberation, which aims “to enable responsible, critical-reflexive decision-making skills” [22], supplements these two methods. This should enable the participants to deal with different arguments and alternative positions, considering inter- and intra-disciplinary controversies, to be able to justify their own position as well as possible in ethical case discussions [23]. Participants analyze the moral dilemma, underlying ethical principles and possible communicative hurdles and assess the situation using the principle-oriented case discussion scheme as per Marckmann [23]. Every principle-oriented case discussion takes place discursively against the background of the discourse ethical approach according to Kessler [24] (for the integration of principle and discourse ethics see [25]). This is not only intended to guarantee equality for all participants in the meeting, regardless of the training stage and the relationship to the case brought in. Rather, the participants learn procedural rules for conducting a discourse with the aim of reducing hierarchies and thus power imbalances [25]. According to the Ulm model of ethics seminars [24], the role of the teacher is primarily that of a moderator who provides information on ethical or legal aspects upon request. The mutual, equal recognition in the context of the discourse and the safe space of the small group enable, in addition to the acquisition of discursive skills, an additional development of the willingness for self-reflection. Furthermore, the discourse continuously addresses personal and social-communicative competencies [26]. 

Last but not least, the case discussions should offer the participants a framework to address the personal perception of moral stress in the respective situation to discuss it within the peer group and to receive support from it and/or the teacher in these situations. 

Pillar 2 (societal medical ethics issue): Problem-based learning and experience-based learning play a key methodological role in pillar 2. Pillar 2 opens the focus to socially relevant and current ethical issues in medicine. To summarize, the participants are addressed less in their role as medical students or doctors, but rather as participants in the socio-ethical discourse. This opens up an ethical topic discussion that deviates from pillar 1, in which the teacher can also take on a more active role if necessary. On the one hand, these learning units underline the current relevance of medical ethics issues beyond the micro level and are intended to give the participants an insight into the broad professional field of medical ethics.

Pillar 3 (lectures by external speakers): The third pillar is used for knowledge transfer and discussion of project-relevant aspects by recognized experts such as on the subject of moral stress. Moreover, this should make the project visible to the outside world (in the clinic and university) and recruit new participants.

#### 2.4. Evaluation

**Figure 1:**
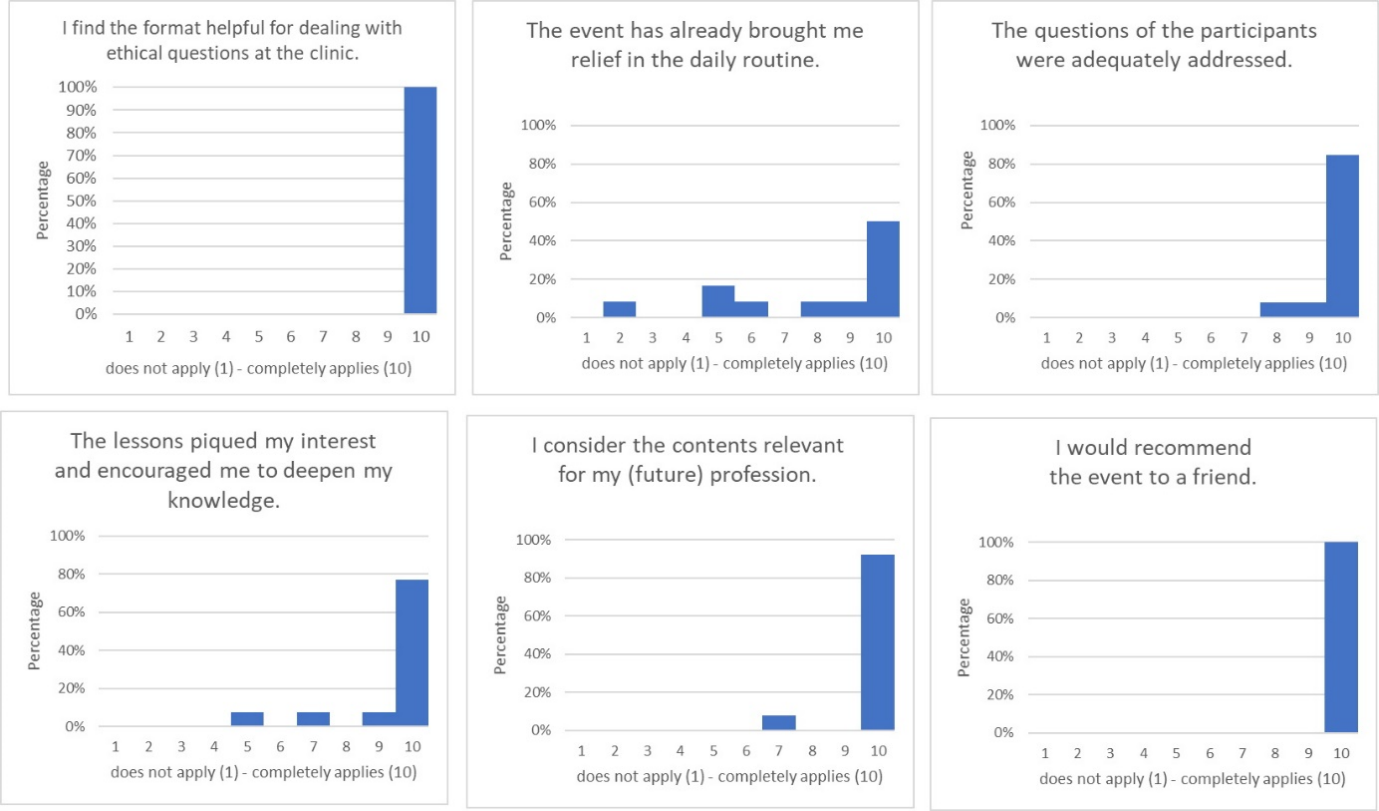
Presentation of the evaluation results

The project management evaluated the project for the first time in January/February 2020 using a self-developed, web-based short evaluation sheet. Therefore, the results presented in chapter 3 and figure 1 (Fig. 1) are a snapshot from the corresponding period. The evaluation questionnaire comprises six closed items, which can be answered with a Likert scale from 1 (does not apply at all) to 10 (fully applies) and with “no answer”. The short evaluation ends with two free text fields in which participants can express what they rate as particularly positive and what should be improved.

The aim of this first, low-threshold, anonymous evaluation round is therefore to derive changes and identify potential for improvement. 

## 3. Results

Only a round three to eight students/newcomers to the medical profession took part in the group discussions (pillars 1 and 2) such that the group size of ten people previously defined as the maximum number of participants was never reached. The group composition remained relatively constant over the project duration with little fluctuation. Around 30 participants from the clinic and medical faculty attended the lectures by external speakers.

 A web-based brief evaluation was performed between January 18 and February 6, 2020. The short evaluation form was sent by e-mail to 17 participants who had voluntarily given their e-mail address to the management of Ethik First or was filled in by 13 participants. The response rate was 76%. These included four students, three PY students and five assistant doctors. One person gave no indication of their level of education.

Figure 1 (Fig. 1) shows the range of answers for the closed questions. Participants submitted a total of twelve free-text comments. Two comments resulted in specific suggestions for improvement, namely, with regard to the project’s previously lacking awareness level and the request for a more detailed theoretical input on different argumentation methods and/or support. The participants cited the network diagram used for decision-making for the extended intensive care treatment requirement for organ donation as an example [18].

The free text answers to the question of what the participants rated particularly positively at Ethik First can be clustered into three main topics:

1. framework conditions,
2. dealing with each other and with the case, and
3. classification in the larger context.

Regarding the framework conditions (1) they appreciated the “good group size” and the fixed duration of one hour per case conference. (2) Participants mentioned the open atmosphere and the open interaction with one another and with difficult issues five times. Ethik First took place in an atmosphere "in which one dares to ask questions" and can “shed light on the case from all sides”. They highlighted the culture of open discussion positively three times. With a view to the larger context (3) the free text comments rated particularly positively that “cases from one's own clinical routine can be brought in” and that Ethik First deals with topics “that are neglected in the course of studies”. Finally, they positively emphasized that Ethik First addresses and shows the “topicality and importance of ethics in relation to [the] rapid progress in research and treatment”.

## 4. Discussion

The interdisciplinary project presented complements the curricular teaching in medical ethics and, in addition to conveying learning content, pursues the aim of addressing moral stress when entering patient care. 

The interim evaluation for a relatively small group of 13 people demonstrates that the project has been rated very positively to date. The free text evaluation shows that the participants have experienced a guided individual case discussion and ethical analysis of cases they have experienced themselves in a small group as a positive enrichment of the mandatory curriculum.

The voluntary nature of the project with a lack of extrinsically motivating factors can partly explain the low attendance rate of three to eight students and young doctors per session. Finally, “the majority of students only consider activities important […] if they are assessed in some way” [27]. The in-depth knowledge and the attitude training in Ethik First have no direct relevance for the state examinations or the specialist examination; thus, there is a risk of them getting lost Issuing certificates should be discussed in this regard to increase motivation. Moreover, it should be considered how the project could reach those who do not identify themselves as interested in ethical issues but who experience moral stress in their training stage and could therefore benefit from support.

The access to the “hidden” and “informal curriculum” of everyday academic and clinical life, obtained through the students' narratives, makes it possible to get closer to the causes of moral stress than through seminars on ethics or ethics consultations or visits [28], [29]. The personal union between the management of the CEC and the management of Ethik First already makes it possible at this point to feed the narratives of the young professionals into the work of the CEC in an anonymized and abstract form and thus to initiate an improvement at the structural level. We envision a widening in the direction of medical history and ethnology for the critical contextualization and analysis of structures, “cultures” and values.

Moreover, we will prioritize the networking of the participants with one another in future and explore opportunities for peer-to-peer mentoring as part of the project’s additional development. 

However, both developments clarify that Ethik First, as an extracurricular offer, is only one component in addressing moral stress. To date, there has been no overarching strategy that considers both the university and the clinical setting, their organization and structure as a whole. 

Against the background of the diverse and growing ethical implications in medical care, from our point of view there is a desideratum of corresponding advanced education concepts both in medical studies and in medical further education. An expansion and development of a project comparable to the three-pillar concept presented here, e.g., at the state medical associations with student participation, should therefore be discussed at a scientific, educational and political level. 

We cannot yet prove the importance of the project for achieving the formulated learning goals and the perception, processing and handling of moral stress. As part of the evaluation, to date only individual and unstructured documented statements have been made that Ethik First has brought relief to the participants in everyday life. For example, we have not yet used the “moral distress thermometer” [30] instrument developed for nursing care in this project. Such a review is made more difficult by the interdisciplinary unresolved question as to what extent - and whether at all - the influence of regular participation in events with an ethical focus (here: Ethik First) for the experience of moral stress, but also the further development of social-communicative and personal competencies can be verified and proven [31], [32]. Schulz et al. have already shown, however, that continuous ethics instruction in small groups in particular has a positive effect on the willingness of doctors to act in moral dilemma situations [33]. As a limitation, it should be mentioned that predominantly those already interested in the subject participated in Ethik First as a voluntary, extra-curricular offer. It can therefore be assumed that there will be a bias in the evaluation.

## 5. Conclusion

Ethik First strengthens the role of ethical aspects in the training of (prospective) doctors and focuses on reflecting on cases they have experienced themselves. The project offers a space to address moral stress and value conflicts, but moves away from small group work because of the three-pillar concept with public events and focuses on societal ethical issues in medicine. First evaluation results show that the support offered is perceived very positively by the participants and that they have benefited from it subjectively. In the long term, Ethik First should be firmly anchored in the university and clinical context, at the interface to the CEC and as a constant in the transition from medical studies to PY and finally when becoming an assistant doctor. 

We formulate a desideratum of corresponding advanced training concepts both in medical studies and in advanced medical training.

## Acknowledgements

This project has become possible thanks to the support of the “UKSH Gutes Tun”-Foundation. 

## Competing interests

The authors declare that they have no competing interests. 

## Figures and Tables

**Table 1:**
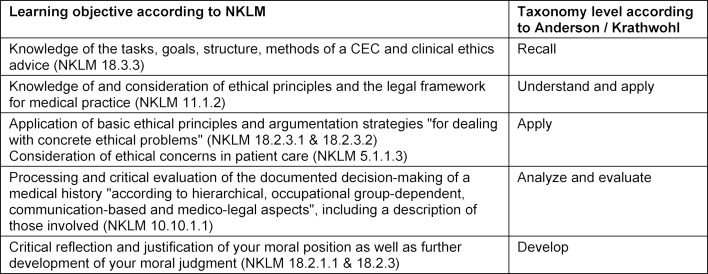
Assignment of learning objectives according to NKLM and taxonomy levels
